# Barriers to Chronic Disease Healthcare Access in Rural Eastern Cape Province, South Africa

**DOI:** 10.3390/ijerph22121881

**Published:** 2025-12-18

**Authors:** Siphelele Mntungwana, Ntiyiso Vinny Khosa, Andiswa Esethu Buso, Nomfuneko Sithole

**Affiliations:** 1 Department of Public Health, Faculty of Medicine and Health Sciences, Walter Sisulu University, Mthatha 5117, South Africa; 221206884@mywsu.ac.za (S.M.); nkhosa@wsu.ac.za (N.V.K.); abuso@wsu.ac.za (A.E.B.); 2 WSU Institute for Clinical Governance and Healthcare Administration, Faculty of Medicine and Health Sciences, Walter Sisulu University, Mthatha 5117, South Africa

**Keywords:** access, barriers, chronic disease, healthcare

## Abstract

**Background**: Inadequate access to healthcare in rural areas worsens the burden of disease, leading to increased morbidity and mortality rates. This study aims to explore the multifaceted barriers that hinder access to chronic disease management at selected rural facilities of the Eastern Cape Province. **Methods**: An exploratory descriptive qualitative design was used to collect data from a sample size of 32 participants (23 patients and 9 health professionals) between November and December 2024. A convenient sampling technique was used to select participants for the study. Semi-structured interviews were audio-recorded; transcribed; and analyzed using thematic analysis. The interview tool was piloted in one of the primary healthcare facilities in the Eastern Cape, which was not used in the actual study. Ethical principles were adhered to throughout the study. The study adhered to the Access to Healthcare conceptual framework. **Results**: The study identified three themes: barriers of access to healthcare services, experiences and perspectives, and existing strategies or interventions. **Conclusions**: Addressing systemic issues like workforce shortages, infrastructure deficits, and socioeconomic challenges in rural Eastern Cape requires a multifaceted approach, including strengthening community-based services and ensuring equitable resource distribution.

## 1. Introduction

Healthcare access is widely recognized as one of the many social determinants of health (SDH), alongside education, income, housing, and employment, which collectively shape health outcomes and equity [1]. Importantly, healthcare access is determined by a range of individual, structural, and systemic factors [2]. Individuals with low socioeconomic status, lack of education, and lack of health literacy are reported to face more challenges with healthcare access [3]. These challenges may include limited mobility, awareness about healthcare systems, and failure to recognize the value of preventative measures such as screening and honoring checkup dates [4]. The availability and accessibility of healthcare and facilities are structural issues. According to the literature, structural issues such as geographic space between the place of residence and points of care, access to the local hospital or clinic, and lack of transportation for patients to visit healthcare centers limit access to healthcare [5,6]. Geographic location, population density, and the allocation of healthcare resources within a specific area might affect the availability and accessibility of healthcare and facilities [7]. Government laws and regulations, healthcare expenditures, and healthcare systems are aspects of systemic factors. Systemic factors can have an impact on healthcare access by determining who is eligible for specific programs or services and how those programs are delivered [2]. Healthcare funding can influence access to healthcare by deciding who can and cannot afford to pay for care.

Access to healthcare is therefore both a social determinant of health and a key mechanism through which other determinants such as education, income, and transport influence health [8]. Despite being a critical determinant of health outcomes, access remains a persistent challenge for millions living in rural areas [8]. Access to healthcare is a fundamental human right and a pillar of any country’s sustainable development. However, rural residents face a variety of challenges in accessing healthcare across the world [1]. The Geneva report by the International Labour Organization shows that 56% of rural residents lack access to essential healthcare services. Literature also reveals that the growing burden of chronic disease in Sub-Saharan Africa exposes weaknesses in healthcare systems [9]. According to McIntyre et al., the system often lacks essential supplies and equipment and faces challenges in allocating resources fairly among different population groups, leaving many individuals without adequate care [9]. Chronic diseases such as diabetes, hypertension, cardiovascular disease, and HIV/AIDS have become leading contributors to morbidity and mortality in countries like South Africa, with rural populations often facing significant barriers to receiving appropriate treatment [10]. As rural communities continue to experience disproportionately high rates of chronic illness and related complications, understanding and addressing the multifaceted obstacles to care is vital [11].

While South Africa has made significant strides in developing healthcare infrastructure and policies to manage these conditions, such as National Health Insurance (NHI), District Health Systems (DHS) and Primary Health Care Re-engineering, profound disparities persist particularly in rural regions like the Eastern Cape (EC) Province with the burden of prevalent non-communicable diseases continuing to rise [12,13]. The EC Province, marked by high poverty levels, limited infrastructure, and a largely dispersed population, presents unique challenges to effective healthcare delivery [14]. In rural EC Province, individuals living with chronic disease face numerous barriers to access consistent and quality healthcare. These include long distances to health facilities, inadequate transportation, shortages of medical staff and medications, cultural differences, and structural limitations within the healthcare system itself [15]. Compounding these factors are social determinants such as education levels, employment status, and traditional health beliefs, all of which influence healthcare-seeking behavior and adherence to treatment regimens [16].

There is limited significant contextual, observed data on the specific barriers that hinder access to chronic disease healthcare in underserved communities. Most existing research on healthcare access in South Africa tends to focus on urban areas or broadly defined rural populations, without adequately capturing the unique socioeconomic challenges faced by rural residents. Moreover, there is limited integration of patients’ perspectives, healthcare professionals’ insights, and contextual factors that influence health-seeking behavior and service delivery for chronic disease in the EC Province. Therefore, this study seeks to explore and analyze the multifaceted barriers that hinder access to chronic disease healthcare services in the EC Province. Barriers that restrict access to healthcare services for chronic disease management in three selected rural facilities (2 clinics and 1 hospital) of the OR Tambo district in the EC Province will be explored, and strategies will be proposed to enhance healthcare access and inform more equitable interventions tailored to the specific needs of these underserved communities.

## 2. Materials and Methods

### 2.1. Context and Conceptual Framework

A qualitative, exploratory-descriptive design was undertaken from 9 to 17 December 2024. The study was conducted at two administrative areas in the OR Tambo district of the Eastern Cape Province. These specifically included purposively selected Community Clinic in Mqanduli, Libode Clinic, and St Barnabas Hospital in Libode to attempt to achieve diversity in participant selection. OR Tambo is one of the province’s eight districts with a population of 1.5 million people, and about 6.8% are over 64 years old [17]. The district occupies the eastern coastal portion of the province, covers an area of approximately 12,141 square kilometers, and incorporates several former Transkei magisterial districts into five local municipalities: King Sabata Dalindyebo, Nyandeni, Port St Johns, Ingquza Hill, and Mhlontlo local municipalities. The OR Tambo district is primarily rural, with agriculture and small subsistence farming being the primary activities. Residents of the district face a considerable number of socioeconomic challenges, such as extreme poverty rates, unemployment, as well as poor infrastructure, health, and education. Ataguba et al. observed that this area of the Eastern Cape Province faces significant economic challenges and is the poorest area in South Africa. Furthermore, disparities in healthcare access remain a pressing concern, with little progress made since 1994 [18].

**Figure 1:**
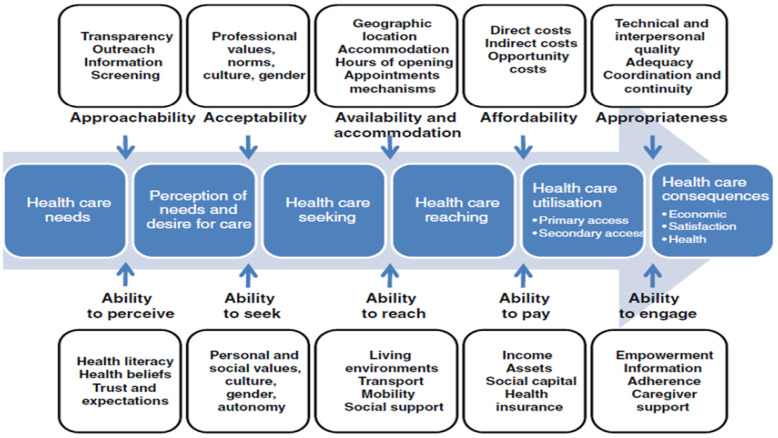
Conceptual framework of access to healthcare. Reprinted with permission from Ref. [20]. Copyright 2013 Levesque, J.F., Harris, M.F. and Russell, G.

A literature review was first performed, looking at the barriers that hinder access to healthcare services for chronic disease management in rural areas of South Africa. Based on the review, access to healthcare was identified for further analysis. This study identified and adopted a conceptual or theoretical framework for access to healthcare as a structured way to understand the multiple factors influencing whether individuals can obtain needed medical services [19]. One of the most widely used frameworks (Figure 1) is one by Levesque et al., who conceptualize access to healthcare across five dimensions that define the ability of individuals to obtain and appropriately use health services [20]. The framework includes availability of necessary services, accessibility regarding geographic proximity and transportation, care affordability, acceptability, and appropriateness to patient needs [20]. Healthcare access also includes chronic disease management needs, which include ongoing care, medication adherence, health education, monitoring, and lifestyle support for conditions such as diabetes, hypertension, and asthma [21]. Among the five dimensions that define the ability of individuals to obtain and appropriately use healthcare services are the five corresponding abilities of patients that could be barriers.

The Levesque et al., conceptual framework’s five corresponding abilities of patients that could be barriers to accessing healthcare were used to structure interview questions and organize findings. Each ability was used as a lens through which barriers were explored and analyzed [20].

#### 2.1.1. Barriers to Perceiving Need (Accessibility)

This is often the first step in a patient’s journey to pursuing care and is determined by individual knowledge, basic health literacy skills, and any existing cultural beliefs about health and illness. The accessibility of health services refers to the provider’s efforts to make their services more known to the patients. It is often related to transparency, outreach, provision of information, and education about services to communities [20].

#### 2.1.2. Barriers to Seeking Care (Acceptability)

Seeking healthcare relates to cultural and social factors affecting services. For acceptability, services must not conflict with patients’ personal, social, or cultural values. The ability to seek health or social care also relates to individual autonomy and the capacity to seek care [20]. Patients with chronic conditions may be able to seek care on their own, while others may perhaps need a family member or a professional (social) care worker.

#### 2.1.3. Barriers to Reaching Healthcare (Availability)

In this dimension, the patients’ ability to physically access services is included, based on personal mobility and external circumstances. In the context of chronic care, the availability of services also refers to the general presence of health services and providers needed and their accessibility to patients, both physically and promptly [20].

#### 2.1.4. Barriers to Utilizing Healthcare and Barriers to the Ability to Pay (Affordability)

According to Levesque et al., affordability refers to patients’ economic capacity to spend resources and time to use the required services, cost, and opportunity costs [20]. The ability to pay for healthcare refers to patients’ economic capacity to pay for healthcare services. In the context of patients with chronic conditions, who usually show higher healthcare utilization rates [22]. Utilizing care particularly includes co-payments and waiting times.

#### 2.1.5. Barriers to Ability to Engage (Appropriateness)

Health outcomes depend on the appropriateness of care and patients’ ability to engage in healthcare. Appropriateness of care is reflected in the suitability between treatment and patients’ needs, timeliness, coordination, and quality. The patients’ ability to engage in healthcare relates to their participation in decision making and compliance in therapy [20]. In chronic care, the appropriateness of care refers to coordination, continuity of care, and interdisciplinary cooperation.

### 2.2. Population and Sampling

The targeted population was all patients with chronic disease and nurses at the research facilities. The participants were recruited at all outpatient departments to determine barriers to healthcare access for chronic disease management. A convenient sampling technique was adopted to select participants based on availability and willingness to participate in the research. A target sample size was set at 30 overall participants: 20 patients and 10 health professionals, with the condition of data saturation, where the researcher would stop at a point where no new information was collected. Included in the study were patients with prevalent chronic conditions: diabetes, hypertension, and cancer, aged 18–70 years, and primary healthcare professionals (Nurses) who provide care to chronic disease patients with at least 6 months of experience working in rural areas. The selected prevalent chronic conditions were particularly in accordance with the growing burden of non-communicable disease (NCDs) in under-resourced and underserved communities such as Eastern Cape Province [13]. Participants with chronic mental illnesses were excluded.

### 2.3. Data Collection

A predefined interview guide for patients and health professionals, with research questions, guided the interviews to gather in-depth information from participants. Qualitative data were collected through semi-structured one-on-one interviews per the predetermined interview guide. Patient participants were approached while waiting in outpatient areas for administrative processes and medical files, and with the help of healthcare providers, they were motivated to participate in the study. The nurses explained the study’s possible benefits, which are improving services at the facilities. Nursing professionals were approached at their nursing stations. After recruitment, participants were directed into a private room one at a time. Each interview began with the researcher sharing information about the study with the participant and obtaining informed consent by asking the participant to complete and sign the consent form. The participants were alerted that the conversation would be recorded with no patient-identifying information and intended harm, and an audio recording consent was obtained. After obtaining all consents, semi-structured key questions were asked with a focus on the following four areas: (1) Demographic profile, (2) Access to healthcare, (3) Barriers/Challenges, (4) Existing strategies to improve access to healthcare. Interviews were conducted in a language that participants preferred and understood, with explanations provided when necessary, and ensuring that the researcher was culturally sensitive and respectful. Each interview lasted for 20–30 min. Data saturation was determined when no new themes and insights were emerging from subsequent interviews. The researcher monitored for saturation through ongoing thematic analysis of transcripts, where it became evident that additional interviews were yielding repetitive information. This study achieved saturation after 32 interviews, aligning with established qualitative research practices.

### 2.4. Data Processing and Analysis

Data captured in the audiotape during the interviews were transcribed and translated verbatim. The two data sets, one for patients and the other for nurses, were processed manually; no software was used. Categorical data analysis was conducted using the six phases of thematic analysis. The process began with familiarization, where the researcher repeatedly listened to the audio recordings and thoroughly reviewed the transcripts to ensure data accuracy and completeness. This was followed by generating initial codes, in which the data were systematically categorized and organized into meaningful codes. The next step involved searching for themes and sub-themes, during which the codes were examined and clustered into broader thematic categories. In the reviewing themes stage, the researcher ensured that the emerging themes accurately reflected the data. Subsequently, the process of defining and naming themes was undertaken, whereby each theme was refined, clearly articulated, and distinguished to capture a specific aspect of the data. Finally, in producing the report phase, the study’s findings were synthesized and evaluated, highlighting the key lessons learned and summarizing the essence of the concept under investigation. Non-categorical data were extracted from the transcripts, captured in an Excel spreadsheet, and descriptively analyzed to report on frequency and percentages.

### 2.5. Ethics Approval

Permission to conduct the study was granted by the Walter Sisulu University Ethics Committee (Ethics approval number: 115/2024), and for data collection by the National Health Research Database of the Eastern Cape, OR Tambo District, KSD Sub-district, and Nyandeni Sub-district, as well as clinic/hospital Chief Executive officers and Managers as gatekeepers of the research sites.

## 3. Results

### 3.1. Participants’ Information

A total of 32 participants (23 patients and 9 healthcare professionals) were obtained from three facilities (Mqanduli CHC, St Barnabas Hospital, and Libode Clinic). The demographic profile of patients and healthcare professionals is demonstrated in Table 1 and Table 2. Most patient participants were females, *n* = 19 (83%), and *n* = 4 (17%) were males, indicating gendered health-seeking behavior. The results show a predominance of older patients (mean age of 60), with most aged 60–69. Chronic diseases like hypertension and diabetes appear to be more prevalent in this age group, emphasizing the need for targeted interventions. Healthcare providers were 9 (28%) and were younger (mean age 33.8 years), suggesting that rural health services rely on the younger workforce. Most were females, consistent with the global trend of women dominating nursing.

#### 3.1.1. Theme 1: Barriers to Accessing Healthcare Services

Participants shared struggles they face when accessing healthcare services for chronic disease management. Barriers reported by patients showed four sub-themes: geographic (accessibility) and infrastructure (availability) barriers, challenges due to transport (appropriateness), economic constraints (affordability), and their impact on access to healthcare.

Below are participant statements describing how difficult it is to travel long distances to reach healthcare facilities, often on poorly maintained roads. One participant stated that this barrier delays treatment, as he cannot go regularly to collect it.

*“Going to the clinic or hospital is difficult because roads are terrible, especially when it rains. Sometimes it takes hours to get to the clinic because taxi drivers refuse to come to our area.”*

[Participant 6, male, 68 years]

*“The clinic is far from where I stay, and the long distance often delays my treatment because I can’t go regularly if it’s raining, or I do not have transport money.”*

[Participant 1, male, 70 years]

*“The hospital is far, and I can’t even walk; I would arrive very late.”*

[Participant 9, female, 67 years]

*“The roads in our area are very bad, especially when it is raining. This makes it difficult even for transport to reach us.”*

[Participant 20, female, 39 years]

*“The roads in my area are very bad, and when it rains, they become muddy and almost impossible to use. This makes it difficult for taxis to operate and for me to reach the clinic.”*

[Participant 26, female, 67 years]

*“Accessing healthcare services has been a struggle for me because of the long distances to the clinic and the lack of transport.”*

[Participant 26, female, 67 years]

Below are participant statements describing how the long distance between patients’ homes and healthcare facilities, combined with poor infrastructure, results in costly and unreliable transport. One participant highlighted the impact of health problems that result from missing appointments.

*“Transport is expensive, so many patients walk long distances.”*

[Participant 21, male 28 years]

*“Patients usually miss their appointments, leading to health issues.”*

[Participant 21, male, 28 years]

*“There is lack of transportation, and our road is a gravel road, making it difficult to travel.”*

[Participant 12, male, 68 years]

*“Transportation is the major issue because I use public transport, and it is not always reliable because when it is raining taxis refuse to reach certain areas.”*

[Participant 20, female, 39 years]

Below are participants’ statements on how socioeconomic constraints impact healthcare access and patient outcomes, resulting in delayed treatment and poor adherence to care plans, as well as the need for strategies to reduce the financial burden on individuals in rural areas. Two participants highlighted “interrupted care” because of economic challenges.

*“This grant is not enough to cater for all my needs, and sometimes it is finished, and I struggle going to the clinic because I also use it for transport.”*

[Participant 2, female, 58 years]

*“The government grant is not enough to cover all my healthcare expenses, including transport and emergencies.”*

[Participant 28, female, 68 years]

*“Yes, it does affect me because I use the same money to buy groceries and pay stokvels, and it usually runs out.”*

[Participant 7, female, 60 years]

*“The money I have is not enough to cover healthcare costs, especially transport.”*

[Participant 27, female, 39 years]

*“Patients experience poor health outcomes due to interrupted care.”*

[Participant 25, male, 25 years]

*“Financial problems patient face usually compromises their treatment adherence.”*

[Participant 30, female, 39 years]

*“It can be difficult sometimes for me to come to the clinic because if I do not have money, I will not be able to come unless I have someone to borrow the money from and come here. Even if it is not my date to collect my treatment then it happens, I get sick, it will be difficult for me because most of the time I don’t have any money left for emergencies.”*

[Participant 5, male, 70 years]

#### 3.1.2. Theme 2: Experiences and Perspectives

Reported experiences and perspectives showed eleven sub-themes which included long waiting times and facility overcrowding (availability), patient dissatisfaction and impact on care (acceptability), medication and resource availability (availability), impact of stock-outs on patients, healthcare worker attitude and behavior (affordability), reliance on alternative remedies and cultural beliefs (acceptability), and perception of traditional medicine (appropriateness). Additionally, the findings highlighted existing and potential strategies to address challenges, improve access to care, and enhance overall healthcare utilization for individuals in rural areas. These insights provide a foundation for developing actionable recommendations to address the identified challenges and promote equitable healthcare access.

Below are participants’ statements on how overcrowding and long waiting times frustrate participants and how this impacts their conditions. High patient-to-staff ratio and limited facilities were viewed as the root cause.

*“Most of the time, I wait for three to four hours, sometimes from 8 a.m. to 1 p.m. This impacts my blood pressure management as I stay here until I am hungry due to time constraints.”*

[Participant 2, female, 58 years]

*“I wait for about three or four hours; it does affect me because I wait here till it is late, and I don’t even have money to buy food.”*

[Participant 7, female, 60 years]

*“It usually depends on how many people are at the hospital. Sometimes I wait for about five or six hours. My diabetes makes me hungry and thirsty all the time.”*

[Participant 15, female, 69 years]

Healthcare professionals’ statements consistently identified long waiting times as the biggest challenge to healthcare access, leading to different consequences.

*“Patients become frustrated and sometimes leave without receiving care.”*

[Participant 21, male, 28 years]

*“It delays diagnosis and early treatment, leading to the chronic disease being worse.”*

[Participant 22, female, 37 years]

Participants’ statements expressing concerns with frequent stock-outs of essential medications at healthcare facilities.

*“Yes, sometimes they say some medication is not available, but I always get my hypertension treatment.”*

[Participant 10, female, 51 years]

*“I usually receive my medication, but this month, they did not give me my diabetes medication, and they did not explain why.”*

[Participant 14, female, 55 years]

*“Yes, I was sent to another clinic and used my own money to get there.”*

[Participant 28, female, 63 years]

*“Insufficient resources at the hospital makes it hard for us to provide comprehensive care to patients.”*

[Participant 24, female, 29 years]

*“I usually receive my medication and sometimes they even give me treatment for three months.”*

[Participant 14, female, 55 years]

All healthcare professionals were asked if there are any medication shortages in their facilities, they all had the same response.

*“Yes”*

[Participant 21–25 and 29–32]

Below are participants’ statements on the impact of Stock-Outs on patients.

*“I was sent to another clinic and used my own money to get there.”*

[Participant 28, female, 63 years]

*“The stock-outs of insulin usually result to mossed doses and lead to poor management of diabetes”*

[Participant 31, female, 29 years]

Below are participants’ statements on reliance on alternative remedies.

*“I’m scared of traditional medicine, including drinking and steaming with them, as I am too old for all those things.”*

[Participant 18, female, 58 years]

*“I used an herb called ‘uzifo zonke’ as I heard it does help with lowering blood pressure levels.”*

[Participant 3, female, 66 years]

*“I used traditional medicine because I heard it also helps in treating diabetes, but I would go to the clinic and still find that my blood sugar is still high.”*

[Participant 28, female, 63 years]

Below are participants’ statements on cultural beliefs and perceptions about traditional medicine.

*“I’m a traditional man. Of course, I am going to use traditional herbs because I believe in them, even my ancestors were using them”*

[Participant 12, male, 68 years]

*“I have used traditional medicines, but they have not been effective in managing both my disease.”*

[Participant 28, female, 63 years]

Below are participants’ statements about healthcare workers attitude and behavior.

*“I have never come across a nurse that was rude to me. I’ve always had the best experience with them, but I think they should try to work fast because we wait for too long here.”*

[Participant 3, female, 66 years]

*“The nurses take care of us here, and they are respectful and kind.”*

[Participant 8, female 64 years]

*“At the hospital, some healthcare workers are respectful and helpful, but I have also encountered others who seem impatient.”*

[Participant 20, female, 39 years]

*“There is limited healthcare staff while there is a large number of patients we have to serve.”*

[Participant 30, female, 29 years]

Below are participants’ statements on the impact of social and safety concerns on women traveling to and from healthcare facilities.

*“It affects because when it is raining, I must hire a ‘special’ to help me cross the river, the place is also not safe for us as women.”*

[Participant 8, female, 64 years]

*“It is very far because I cannot even walk to the hospital or clinic, and it is not safe for women to walk alone in the street.”*

[Participant 18, female, 58 years]

Below are participants’ statements on workplace challenges in providing chronic disease management in rural healthcare settings.

*“Heavy workload and still have to provide quality care for patients.”*

[Participant 21, male, 28 years]

*“Emotional strain that comes from seeing a patient suffer and sometimes we cannot even help them due to insufficient medical supplies.”*

[Participant 25, male, 37 years]

Below is a participant statement about delayed patient access to care.

*“Patients usually arrive late at hospital when their condition has progressed because ambulances do not arrive on time making it harder for us.”*

[Participant 21, male, 28 years]

Below are participants’ statements about the shortage of staff, heavy workload, and burnout among healthcare workers.

*“Overwork and burnout are the most challenges here at the hospital because it is always overcrowded and sometimes, we do not even get our lunch breaks”*

[Participant 22, female, 37 years]

*“Heavy workload due to shortage of staff here at the clinic.”*

[Participant 29, female, 30 years]

#### 3.1.3. Theme 3: Existing Strategies or Interventions

Participants recognized efforts such as mobile clinics and Community Health Worker (CHW) Programs with medication pre-packaging as strategies or attempts to increase access to healthcare services. However, they noted that these interventions had successes and challenges as they were sometimes inconsistent, especially with mobile clinics.

Below are participants’ statements about mobile clinics and CHW Programs.

*“The only thing I have seen are mobile clinics even though they do not come here regularly.”*

[Participant 1, male, 70 years]

*“I have seen mobile clinics occasionally and NGOs in the community, but the NGOs focuses on children only.”*

[Participant 5, male 70 years]

*“Mobile clinics do come occasionally, because it is also difficult for them to come more often because our roads are in a poor condition.”*

[Participant 6, male, 68 years]

*“We do have mobile clinics even though they often reach areas far from where I stay, so I wouldn’t know what they offer because I have never gone to them.”*

[Participant 7, female, 68 years]

*“Mobile clinics sometimes come to the school, but they don’t come regularly. When they do come, they give people medication for their illnesses and check blood pressure and diabetes.”*

[Participant 9, female, 67 years]

*“The CHW’s we had used to visit children and even give them deworming medicine.”*

[Participant 9, female, 67 years]

*“I have only seen mobile clinics, but they take a month or two to come back again.”*

[Participant 10, female, 51 years]

*“They only visit those who cannot go to the clinic on their own, they bring them treatment.”*

[Participant 16, female, 68 years]

*“Once in three months. Their visits are helpful, but not consistent enough to manage my condition.”*

[Participant 27, female, 39 years]

*“Mobile clinics have been effective in reaching many patients in rural areas, but the lack of resources, such as staff and equipment has made it difficult to reach even more people.”*

[Participant 21, male, 28 years]

Below are participants’ statements about the success and challenges of community interventions.

*“Outreach programs help in raising awareness and detection of chronic disease at an early stage, the only challenge is that there is not enough staff to be part of the team.”*

[Participant 22, female, 37 years]

*“Outreach programs and mobile clinics. It is often difficult to reach some communities due to poor infrastructure and lack of staff.”*

[Participant 25, female, 37 years]

## 4. Discussion

Despite global and national efforts to improve healthcare access, including efforts to achieve Universal Health Coverage [23], this study highlights the persistent barriers that patients in rural areas face when trying to access healthcare services for chronic disease management. These barriers go beyond financial constraints, geographic accessibility of healthcare facilities, and systemic challenges that patients and healthcare providers face. The findings of this study shed light on participants’ demographics, structural, cultural, and broader social determinants of health (SDOH), which show inequalities specifically in rural healthcare, within which healthcare access itself is a critical determinant [16].

The participants’ demographic profile showed the characteristics of patients and healthcare workers in rural areas. The predominance of female patients (82%) accessing healthcare for chronic disease shows a gendered health-seeking behavior. Studies indicate that women are more proactive in seeking healthcare than men [22,24]. This trend suggests that targeted interventions are needed to address cultural norms and barriers preventing male participation in chronic disease management. The high prevalence of older adults among the patient population (mean age of 60 years) is consistent with global trends showing that as people age, they are more likely to have chronic disease [25]. Chronic diseases such as hypertension (56%) and diabetes (12%) were particularly observed in this study for individuals aged 60 years and above, which is reported to be common [26]. These older populations are particularly susceptible to inequalities in healthcare access as a social determinant of health [27], underscoring the need for age-sensitive interventions.

The Levesque et al., framework guided the review of barriers to healthcare and provided useful insights into the study population [20]. The findings highlighted key structural challenges in rural healthcare facilities, including inadequate staffing, poor infrastructure, and limited resources, consistent with existing literature on rural health access [28]. Participants reported shortages of medical supplies, long waiting times, and staff burnout due to heavy workloads, all of which compromise the quality of care. The findings underscore the need for greater investment in infrastructure and human resources, alongside robust monitoring and evaluation systems to track the impact of interventions. From a social determinants of health (SDOH) perspective, these barriers reflect structural and systemic inequities that shape healthcare access. Multidisciplinary models that integrate doctors, nurses, social workers, community health workers, and mental health specialists have been suggested as strategies to strengthen service delivery and reduce the burden on staff [7]. Such models could improve the efficiency and quality of care provided in rural healthcare facilities.

Cultural norms and beliefs were identified as influential in a patient’s health-seeking behavior. Many participants reported a reliance on traditional medicine and home remedies, sometimes due to cultural beliefs, affordability, and easy access. However, others noted that while traditional medicine was easily accessible and affordable, they were not always effective in treating or managing chronic disease. This reliance on traditional medicine aligns with the findings from studies conducted in other rural settings, where cultural beliefs and lack of knowledge often contributed to delays in seeking medical care [29]. However, studies such as Henke et al., argue that reliance on traditional medicine may decline as formal healthcare systems improve and become more accessible for individuals [30]. The results highlight the need for culturally sensitive health education programs that address misconceptions about chronic disease management and promote the integration of traditional and biomedical approaches in rural areas. However, this needs more community health workers. The study findings revealed a shortage of community health workers in rural areas for conducting these education programs. Some participants said that even the ones that are available need adequate training.

A wide range of social determinants of health, such as income, transport, and education, were highlighted as critical barriers to healthcare access. Participants often mentioned excessive travel costs as a significant challenge, a finding consistent with studies identifying financial barriers as a key factor in rural healthcare inequities [31]. Poor transportation infrastructure intensified the problem, particularly during rainy seasons, and this often forced residents to walk long distances to reach public transport or prevented mobile clinics from reaching the community. While poor road infrastructure is a persistent barrier, community-based programs have been suggested as effective complementary solutions [32]. These findings reinforce healthcare access as both shaped by and interacting with other SDOH, making it a connection point for interventions.

Overcrowding of healthcare facilities emerged as a major issue, with participants reporting long waiting times due to staff and resource shortages. A common observation was that clinics and hospitals serve multiple communities, causing delays in care [33]. Participants also reported frequent shortages of medication and specialized services, requiring costly travel to distant tertiary hospitals. Referrals to these facilities created further financial strain, as patients often had to cover transport and food costs, though ambulances were occasionally available. While these findings align with previous studies [34], others suggest that resource allocation in rural healthcare is improving in some regions [30,34]. Improving healthcare access requires a multifaceted approach that addresses availability, affordability, and quality of services. Key strategies include reducing financial barriers through subsidies or insurance, strengthening infrastructure in underserved areas, expanding telemedicine and mobile health services, and promoting community engagement to improve health literacy [35]. Although mobile clinics and outreach programs were reported by healthcare workers, several patients indicated they had little or no access to such interventions, often due to poor road infrastructure.

## 5. Conclusions

This study explored barriers to accessing healthcare services for chronic disease management in a rural area of the Eastern Cape Province of South Africa. The findings showed three themes: barriers of access to healthcare services, experiences and perspectives, and existing Strategies or Interventions. This study demonstrates that healthcare access, understood as one of the many social determinants of health, is both a determinant and a mediator of other SDOH, such as income, education, and transport. Barriers identified in this study illustrate the complex, intersecting nature of these determinants in rural Eastern Cape Province. Strategies for enhancing healthcare access must therefore adopt a multifaceted approach: reducing financial barriers through subsidies or insurance, investing in infrastructure and human resources, implementing telemedicine and mobile outreach, and integrating culturally sensitive education programs. Strategies and initiatives should strengthen human resources for health, improve equity in healthcare delivery, and ensure sustainability to meet the growing demands for chronic disease management. Policy efforts should prioritize rural equity by addressing systemic and structural determinants, thereby strengthening healthcare access as a pathway to improving overall health outcomes.

While this study has its limitations, the findings offer valuable insight into the experiences of patients and healthcare professionals, especially at the two sub-districts of the OR Thambo district, emphasizing the urgent need for targeted interventions. The study was conducted in two sub-districts of OR Tambo in the Eastern Cape Province. Because the sample size was smaller than expected, the findings can only be partly generalized to the OR Tambo district and not to other districts of the province. However, the results provide valuable insights for improving chronic disease management in rural health facilities across the Eastern Cape. Future studies with larger samples from at least two districts are recommended to better represent the province and to guide intervention strategies. Such interventions should include increased funding for rural healthcare facilities, expanded training and recruitment of healthcare workers, and culturally appropriate health education programs to ensure patients receive comprehensive and effective treatment.

## Figures and Tables

**Table 1:** Patient Demographic Information.

Codes	Gender	Age	Diagnosis	Year of Diagnosis	Facility Name
Participants 1	Male	70	Hypertension	>10 years	Mqanduli CHC
Participants 2	Female	58	Hypertension	2012	Mqanduli CHC
Participants 3	Female	66	Hypertension	>10 years	Mqanduli CHC
Participants 4	Female	50	Diabetes & Hypertension	2020 & 2022	Mqanduli CHC
Participants 5	Male	70	Hypertension	Unknown (>10 years)	Mqanduli CHC
Participants 6	Male	68	Hypertension	Unknown (>10 years)	St Barnabas Hospital
Participants 7	Female	60	Diabetes & Hypertension	Unknown (>10 years)	St Barnabas Hospital
Participants 8	Female	64	Hypertension	Unknown (>10 years)	St Barnabas Hospital
Participants 9	Female	67	Hypertension	2001	St Barnabas Hospital
Participants 10	Female	51	Diabetes & Hypertension	2024	St Barnabas Hospital
Participants 11	Female	64	Hypertension	2002	St Barnabas Hospital
Participants 12	Male	68	Hypertension	2020	St Barnabas Hospital
Participants 13	Female	50	Hypertension	Unknown	St Barnabas Hospital
Participants 14	Female	55	Diabetes & Hypertension	Unknown	St Barnabas Hospital
Participants 15	Female	69	Diabetes	2020	St Barnabas Hospital
Participants 16	Female	68	Hypertension	Unknown	St Barnabas Hospital
Participants 17	Female	67	Hypertension	Unknown	St Barnabas Hospital
Participants 18	Female	58	Hypertension	2020	St Barnabas Hospital
Participants 19	Female	65	Hypertension	2016	St Barnabas Hospital
Participants 20	Female	39	Breast Cancer	2024	St Barnabas Hospital
Participants 26	Female	67	Hypertension	2018	Libode Clinic
Participants 27	Female	39	Diabetes	2022	Libode Clinic
Participants 28	Female	63	Diabetes	2022	Libode Clinic

Total number (*n*) of 19 Females and 4 Males.

**Table 2:** Health professional demographic information.

Code	Gender	Age	Professional Level	Facility Name
Participants 21	Male	28	Registered Nurse	St Barnabas Hospital
Participants 22	Female	37	Registered Nurse	St Barnabas Hospital
Participants 23	Female	40	Registered Nurse	St Barnabas Hospital
Participants 24	Female	29	Registered Nurse	St Barnabas Hospital
Participants 25	Male	37	Registered Nurse	St Barnabas Hospital
Participants 29	Female	30	Registered Nurse	Libode Clinic
Participants 30	Female	39	Registered Nurse	Libode Clinic
Participants 31	Female	42	Registered Nurse	Libode Clinic
Participants 32	Female	28	Registered Nurse	Libode Clinic

## Data Availability

Data supporting reported results is available upon request, a link will be provided.
